# Beneficial Effects of Melatonin in the Ovarian Transport Medium on In Vitro Embryo Production of Iberian Red Deer (*Cervus elaphus hispanicus*)

**DOI:** 10.3390/ani10050763

**Published:** 2020-04-27

**Authors:** Irene Sánchez-Ajofrín, María Iniesta-Cuerda, Patricia Peris-Frau, Alicia Martín-Maestro, Daniela-Alejandra Medina-Chávez, Carolina Maside, María Rocío Fernández-Santos, José Antonio Ortiz, Vidal Montoro, José Julián Garde, Ana Josefa Soler

**Affiliations:** 1 SaBio IREC (CSIC-UCLM-JCCM), ETSIAM, Campus Universitario s/n, 02071 Albacete, Spain; 2 Medianilla S.L. Finca Las Lomas, Vejer de la Frontera, 11179 Las Lomas, Cádiz, Spain

**Keywords:** melatonin, deer, sheep, ovary storage, transport, oocyte, embryo

## Abstract

**Simple Summary:**

The development of in vitro embryo production (IVP) in wild species, such as Iberian red deer, can become a daunting challenge since prolonged ovary transport times to the laboratory are often unavoidable. This may have detrimental effects on the quality and developmental capacity of oocytes. We evaluated the effect of supplementing the ovary transport medium with the antioxidant melatonin and observed an increased level of oocyte intracellular reduced glutathione content. Moreover, melatonin enhanced cleavage and blastocyst rates and had a positive effect on embryo quality in terms of the expression of essential embryo development-related genes. In conclusion, the addition of melatonin to the ovary storage medium could mitigate the negative impacts that long transport times may have on oocyte developmental competence and quality of the resulting blastocysts in Iberian red deer.

**Abstract:**

A major limiting factor for the development of in vitro embryo production (IVP) in wild species, such as Iberian red deer, compared to livestock animals is the poor availability and limited access to biological material. Thus, the use of post-mortem ovaries from slaughtered animals represent a source of oocytes for the large scale production of embryos needed for research and to improve the efficiency of IVP. However, these oocytes are not as developmentally competent as their in vivo counterparts. Moreover, oocytes are usually obtained from ovaries that have been transported for long distances, which may also affect their quality. In order to overcome the issues associated with prolonged storage times of post-mortem material, in this study we examined the effect of melatonin supplementation to the ovary transport medium on oocyte quality, embryo yield, and blastocyst quality in Iberian red deer. When necessary, sheep was used as an experimental model due to the large number of samples required for analysis of oocyte quality parameters. Oocytes were in vitro matured and assessed for early apoptosis; DNA fragmentation; reactive oxygen species (ROS); reduced glutathione (GSH) content, mitochondrial membrane potential, and distribution; and relative abundance of mRNA transcript levels. After in vitro fertilization, embryo rates and blastocyst quality were also investigated. The results revealed that melatonin treatment significantly increased intracellular level of GSH in sheep oocytes. Moreover, the percentage of cleavage and blastocyst yield in red deer was greater compared to the Control group and there was lower abundance of oxidative stress- and apoptosis-related *SHC1*, *TP53*, and *AKR1B1* mRNA transcripts in blastocysts for the Melatonin group. In conclusion, the supplementation of melatonin to the ovary storage medium had a positive effect on the developmental competence and quality of resulting blastocysts in Iberian red deer.

## 1. Introduction

The Iberian red deer (*Cervus elaphus hispanicus*) is a wild subspecies of red deer that only inhabits the Iberian Peninsula. Its genetic value is well-known worldwide and the production of this subspecies is related to the hunting industry for commercial purposes. The use of assisted reproduction technologies, like in vitro embryo production (IVP), in red deer had been previously acknowledged [1,2] and may play an important role for the purpose of improving individuals for trophy hunting and ensuring genetic advancement and avoidance of inbreeding depression as a result of the genetic isolation of wild populations within fenced game estates [2,3]. Additionally, though Iberian red deer is not endangered, its conservation is highly recommended [4].

Unfortunately, the process of developing IVP protocols and improving its efficiency in wild species is a daunting challenge due to biological and technical aspects; it requires large numbers of experimental samples and there is often limited access to the animals of study [5,6]. In that scenario, the use of ovaries of slaughtered animals can be a source of oocytes for the large scale production of embryos through IVP procedures [7]. However, compared to in vivo counterparts, the harvesting of follicular oocytes from dead animals is known to produce less developmentally competent oocytes [8]. In the case of Iberian red deer it is possible to obtain ovaries from animals slaughtered in abattoirs or slaughterhouse-like designated facilities. However, these places are usually far from laboratories. Although in vitro embryos have been successfully produced in this deer subspecies from dead animals [2], blastocyst rates remain low compared to other small ruminants like sheep [9].

After slaughter, the lack of irrigation places the ovaries under ischemic conditions, reducing oxygen and energy supplies [10]. In this situation, the production of reactive oxygen species (ROS) may be increased within the follicle [11], overwhelming the endogenous antioxidant systems, thus leading to oxidative stress and oocyte damage [12,13]. To reduce the harmful effects of ROS in the oocyte, several antioxidants have been used during in vitro maturation (IVM) [14], though their supplementation may be more favorable at an earlier stage. Interestingly, Zhau and Zou [15] showed that a rapid increase in the levels of circulating ROS can occur three minutes after the onset of ischemia in rat cardiac tissue. Moreover, of all adult organs in mammals, female reproductive tissues have some of the greatest rates of blood flow per unit of tissue and also exhibit a high metabolic rate [16]. Therefore, in ischemic reproductive organs, the increase of oxygen free radicals might take place as quickly as in other metabolically “expensive” tissues and organs such as heart, kidneys, and brain [17]. Hence, it is very likely that the addition of antioxidants to the ovary storage medium has a crucial role in germ cell protection, especially when long ovary transport times cannot be avoided.

Antioxidants are helpful for minimizing oxidative stress induced by excessive ROS production by clearing free radicals and lowering ROS levels [18]. Accordingly, melatonin (N-Acetyl-5-Methoxy Tryptamine) is a powerful direct scavenger of free radicals with antioxidant and antiapoptotic properties [19]. The ability of melatonin to protect against oxidative stress has been extensively studied during IVM and in vitro culture (IVC), and has demonstrated a positive effect in different species such as cattle [20,21], sheep [22], mouse [23], pig [24,25,26,27], and rabbit [28]. These beneficial effects have been attributed to its ability to decrease the expression of proapoptotic genes, increase that of antiapoptotic genes, and neutralize ROS [29]. However, there is limited information about the effect of melatonin during long ovary transports. In this respect, Goodarzi et al. [30] showed good results of this antioxidant in terms of oocyte developmental competence and blastocyst quality in sheep. This study also revealed that the storage of ovaries at 20 °C compared to 4 °C adversely affected the ability of oocytes to develop to the blastocyst stage. It is known that temperature, medium composition, and storage time are determinant factors in organ preservation [11,31]. This has been shown in several studies where ovary transport conditions influenced IVP success rates in different species [10,32,33,34,35]. Although the effect of temperature during shipment of ovaries has been examined in Iberian red deer [2], to the best of our knowledge, there are no reports regarding the impact of an antioxidant in the ovary preservation medium in this species.

For the purpose of improving the current poor in vitro fertilization (IVF) outcomes in Iberian red deer, the goal of this study was to evaluate the effects of supplementing the ovary transport medium with melatonin on oocyte quality, embryo production, and blastocyst quality. Due to the low availability of Iberian red deer samples and the great number of oocytes required for analysis of oocyte quality, parameters such as ROS and glutathione (GSH) content; early apoptosis; DNA fragmentation; and mitochondrial membrane potential and distribution were evaluated in sheep since it is a valuable research model [30].

## 2. Materials and Methods

Except where otherwise stated, all chemicals and media were acquired from Merck Life Sciences (Madrid, Spain).

### 2.1. Recovery of Oocytes and In Vitro Maturation

Ovaries were collected from animals slaughtered in slaughterhouse. Handling of those animals followed the Spanish Policy for the care of animals, their exploitation, transport, experimentation, and slaughter (Law 32/2007).

Ovaries of adult sheep and Iberian red deer were collected post-mortem from a local abattoir and a slaughterhouse-like designated facility, respectively, during spring season. These were transported to the laboratory at 30 °C and randomly divided between the two treatment conditions: 8.9 g/L physiological saline and 0.1 g/L penicillin (Control) or 8.9 g/L physiological saline, 0.1 g/L penicillin, and 10^−3^ M melatonin (Melatonin). The stored ovaries from deer and sheep were processed between 7 h and 11 h after slaughter of the animal. To obtain the cumulus-oocyte complexes (COCs), ovaries were sliced with a scalpel in TCM199 medium supplemented with HEPES (2.38 mg/mL), heparin (2 μL/mL), and gentamycin (4 μL/mL). The COCs (sheep = 1227 and deer = 445) were then washed in TCM199 and 4 μL/mL gentamycin, and those encircled by at least three layers of compact cumulus cells and a homogeneous ooplasm were selected and added to four-well dishes containing 500 μL of IVM medium/TCM199 supplemented with 4 μL/mL of gentamycin, 100 μM of cysteamine, 10 mg/mL of FSH, 10 mg/mL of LH, and 10% fetal calf serum (FCS) [2] under mineral oil (Nidacon, Gothenburg, Sweden). Oocytes were cultured for approximately 22 h at 38.5 °C in a humidified atmosphere with 5% CO_2_ in air. After maturation, 133 oocytes in sheep and 76 in deer were collected and mechanically denuded of cumulus cells, snap-frozen in liquid nitrogen, and stored at −80 °C until mRNA extraction and reverse transcription.

### 2.2. Collection of Follicular Fluid and Melatonin ELISA Assay

Follicular fluid from Control and Melatonin sheep ovaries was collected in a sterile Eppendorf^®^ 1.5 mL tube. Follicular fluid was subjected to 15 min of centrifugation at a speed of 3300 rpm at 4 °C. Afterward, supernatant was centrifuged again in the same conditions. The concentrations of melatonin in the follicular fluid were determined by Melatonin saliva ELISA kits (Tecan-IBL International, Hamburg, Germany). All ELISA experiments were run in compliance with the manufacturer’s protocol. Melatonin levels were determined as average levels.

### 2.3. In Vitro Fertilization

Sheep and red deer matured COCs were washed and the cumulus cells were partially removed from the oocytes by gentle pipetting. Sperm samples were supplied by the Germplasm Bank of Universidad de Castilla-La Mancha (UCLM). The Germplasm Bank of UCLM is officially authorized for storing semen (ES07RS02OC) following the RD 841/2011. Frozen straws from two rams and three deer were thawed and spermatozoa were centrifuged over a Percoll^®^ gradient (45%/90%). Selected spermatozoa were capacitated for 15 min at 38.5 °C and 5% CO_2_ in 450 μL of in vitro fertilization (IVF) medium: synthetic oviductal fluid (SOF), as established by Takahashi and First [36], supplemented with 10% estrous sheep serum (ESS) in sheep, and 450 μL of a modified SOF (5 mM CaCl_2_·2H_2_O) supplemented with 20% ESS in deer, as previously described by Sánchez-Ajofrín et al. [9]. Spermatozoa and oocytes were co-incubated under mineral oil (Nidacon, Gothenburg, Sweden) at 38.5 °C in 5% CO_2_, 5% O_2_, and 90% N_2_ air with high humidity. A final sperm concentration of 10^6^ spermatozoa/mL was used for insemination, and groups of approximately 40 oocytes were placed in each well.

### 2.4. In Vitro Embryo Culture

At approximately 18 h after IVF, presumptive zygotes were washed four times and transferred to four-well plates containing 25 μL droplets of IVC medium: SOF supplemented with 3 mg/mL of bovine serum albumin (BSA) under 750 μL of mineral oil (Nidacon, Gothenburg, Sweden). Embryos were incubated at 38.5 °C in 5% CO_2_, 5% O_2_, and 90% N_2_ air with high humidity for 8 days post-insemination (dpi). Cleavage rate was evaluated at 48 h post-insemination (hpi), and blastocyst rate was recorded on 6, 7, and 8 dpi. All expanded blastocysts were washed three times in PBS supplemented with 0.1% *w*/*v* PVA (PBS-PVA) and either snap-frozen and stored at −80 °C until mRNA analysis or fixed in a 100 µL drop of glutaraldehyde solution (4% *w*/*v* in PBS, pH 7.4) at room temperature for cell-number evaluation.

### 2.5. Determination of Early Apoptosis

**Figure 1:**
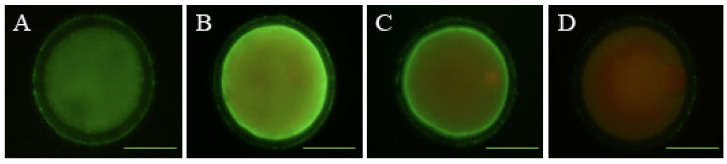
Representative images of early apoptosis detection in sheep oocytes. Oocytes were stained by Annexin V and PI. (**A**) Viable oocytes; (**B**) early apoptotic oocytes; (**C**,**D**) non-viable oocytes. Scale bar = 52 µM.

**Figure 2:**
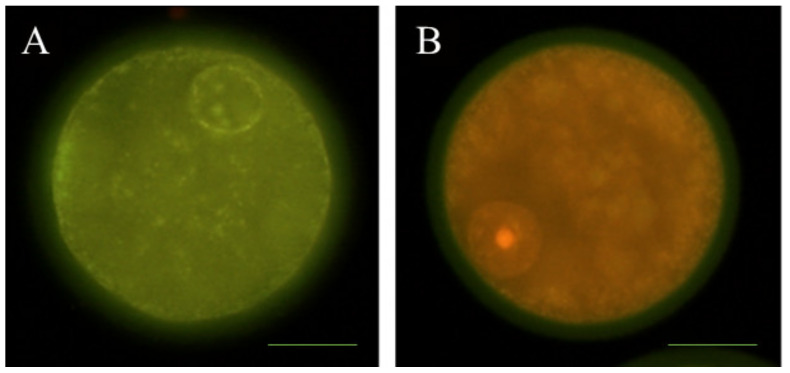
Representative images of DNA fragmentation by TUNEL assay in sheep oocytes at the germinal vesicle (GV) stage. (**A**) TUNEL-positive oocyte; (**B**) TUNEL-negative oocyte. Scale bar = 38 µM.

The detection of early apoptosis in sheep oocytes was performed with the Annexin V, FITC conjugate kit (Invitrogen, Barcelona, Spain) following the manufacturer’s instructions with slight modifications. Briefly, 100 in vitro-matured sheep oocytes were mechanically denuded and placed in 100 µL Annexin V binding buffer droplets containing 5 µL of Annexin V/FITC and 1 µL of propidium iodide (PI; 100 µg/mL) and incubated at 37 °C in the dark for 15 min. Oocytes were counterstained with PI, a membrane impermeable stain, to distinguish between live cells and dead cells. After incubation, oocytes were washed thrice in PBS-PVA, mounted on slides and observed by fluorescence microscopy (Eclipse 80i, Nikon Instruments Europe, Amsterdam, Netherlands). Oocytes were classified into three groups: (1) viable oocytes, negative for Annexin V and PI signal (Figure 1A); (2) early apoptotic oocytes, positive for Annexin V green staining of the ooplasmic membrane and PI negative (Figure 1B); and (3) non-viable oocytes, with Annexin V and PI positive signals (Figure 1C) or negative for Annexin V and positive for PI signal (Figure 1D).

### 2.6. Detection of DNA Fragmentation

Denuded in vitro-matured sheep oocytes (*n* = 93) were fixed in 0.5% *v*/*v* glutaraldehyde for 20 min at room temperature and stored in PBS-PVA at 4 °C before TUNEL assay (Tdt-mediated dUTP nick-end labelling). Fixed samples were permeabilized in 0.5% Triton X-100 in PBS for 1 h at room temperature. Next, positive control samples were incubated with DNAse (0.2 U/µL) at 37 °C in the dark for 1 h. Immediately after, the In Situ Cell Death Detection Kit, fluorescein (Merck Life Sciences, Madrid, Spain) was used for TUNEL staining according to the manufacturer’s instructions. Samples were placed in 30 µL droplets of TUNEL reagent containing fluorescein isothiocyanate conjugated dUTP and the enzyme terminal deoxynucleotidyl transferase (TdT) and then incubated in the dark for 1 h at 37 °C. The negative control was incubated in the absence of TdT. Afterward, matured oocytes were washed three times in PBS-PVA and transferred onto slides in a drop of SlowFade^™^ Gold Antifade Mountant with 6.25 µg/mL PI and 1 μg/mL Hoechst 3342 fluorescent dye, respectively. Samples were evaluated using a fluorescence microscope (Nikon Eclipse 80i). Stages of apoptosis were classified as TUNEL-positive (Figure 2A) and TUNEL-negative (Figure 2B) according to fragmented cell nuclei.

### 2.7. Determination of Intracellular ROS and GSH Levels

**Figure 3:**
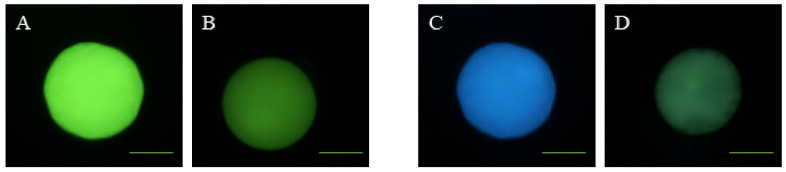
Representative images of reactive oxygen species (ROS) and glutathione (GSH) levels in sheep oocytes. (**A**) High ROS intensity; (**B**) low ROS intensity; (**C**) high GSH intensity; (**D**) low GSH intensity. Scale bar = 51 µM.

To detect intracellular ROS and GSH levels, a total of 133 denuded in vitro-matured sheep oocytes were incubated with 10 µM of 2′,7′-dichlorodihydrofluorescein diacetate (CM-H_2_DCFDA; Invitrogen, Barcelona, Spain) and 50 of µM CellTracker™ Blue CMF_2_HC dye (Thermo Fisher Scientific, Barcelona, Spain), respectively, for 30 min at 37 °C in the dark. After incubation, oocytes were washed thrice with PBS-PVA and placed on glass slides under cover slips. The images were observed with a fluorescence microscope (Eclipse 80i, Nikon Instruments Europe, Amsterdam, Netherlands) and the fluorescence intensity was analyzed by ImageJ 1.45s software (National Institutes of Health, Bethesda, Rockville, MD, USA). Oocytes were classified as having a high (Figure 3A) or low (Figure 3B) ROS intensity and high (Figure 3C) or low (Figure 3D) GSH intensity.

### 2.8. Mitochondrial Membrane Potential Analysis

**Figure 4:**
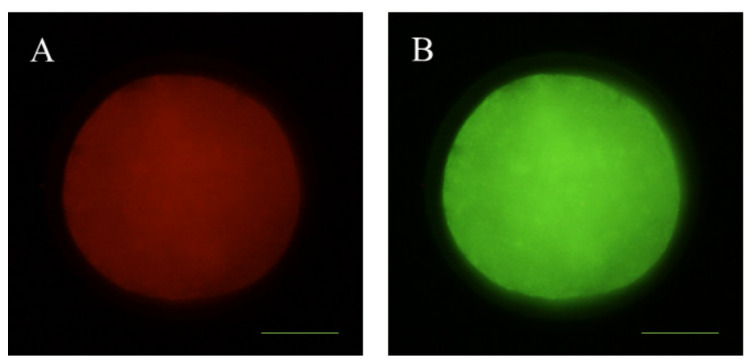
Representative images of JC-1 stained mitochondria in sheep oocytes. (**A**) Mitochondria with high membrane potential (red fluorescence); (**B**) mitochondria with low membrane potential (green fluorescence). Scale bar = 21 µM.

To monitor mitochondrial membrane potential changes, 96 denuded in vitro-matured sheep oocytes were incubated with 2 μM JC-1 dye (5,5′,6,6′-tetrachloro-1,1′,3,3′-tetraethylbenzimidazolyl-carbocyanine iodide (Thermo Fisher Scientific, Barcelona, Spain). All oocytes were stained at 37 °C for 30 min. This reagent is a cell permeant dye that exhibits two emission peaks of green fluorescence and red fluorescence to indicate J-monomers (low mitochondrial membrane polarization) and J-aggregates (high mitochondrial membrane polarization). Relative mitochondrial membrane potential was calculated using ImageJ 1.45 s software as the ratio of red fluorescence (activated mitochondria/J-aggregates; Figure 4A) to green fluorescence (less-activated mitochondria/J-monomers: Figure 4B).

### 2.9. Examination of Mitochondrial Distribution

**Figure 5:**
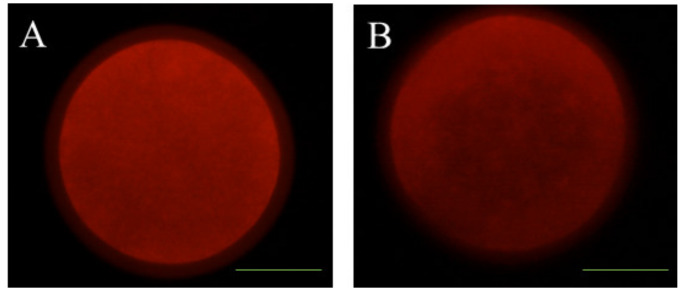
Representative images of mitochondrial distribution of sheep oocytes. (**A**) Red mitochondria normally distributed (homogeneous distribution; Figure 5A); (**B**) red mitochondria abnormally distributed (Figure 5B). Scale bar = 24 µM.

The mitochondrial distribution pattern was examined by MitoTracker^®^ Red CMXRos (Thermo Fisher Scientific, Barcelona, Spain). A total of 100 denuded in vitro-matured sheep oocytes were incubated for 20 min at 37 °C in 100 nM dye. The labeled oocytes were washed twice for 5 min, placed on glass slides, and examined by fluorescence microscopy (Eclipse 80i, Nikon Instruments Europe, Amsterdam, Netherlands). Mitochondrial distribution was categorized into two categories according to cytoplasm oocyte distribution: homogeneous (normal) distribution (Figure 5A) and abnormal distribution (Figure 5B).

### 2.10. In Vitro Maturation Assessment

A total of 182 denuded sheep oocytes were fixed in 0.5% glutaraldehyde (*v*/*v*) for 20 min at room temperature. Subsequently, oocytes were incubated in one drop of SlowFade^™^ Gold Antifade Mountant containing 1 μg/mL Hoechst 33342 in PBS-PVA for 10 min. Fixation and Hoechst 33342 staining procedures were performed at room temperature. After every staining or fixing procedure, oocytes were washed three times in PBS-PVA and mounted on slides sealed under coverslips with vaseline. After 20 min, nuclear configurations were assessed using an epifluorescent microscope (Eclipse 80i, Nikon Instruments Europe, Amsterdam, Netherlands). Chromatin configurations were classified as follows: immature or germinal vesicle (GV), when the vesicle was clearly visible; resumption of meiosis or germinal vesicle break down (GVBD), when the chromatin was dispersed and initiating condensation; metaphase I (MI), when chromosomes became arranged on the metaphase plate and were attached to the meiotic spindle; and mature or metaphase II (MII), when chromosomes were in the second metaphase plate and the first polar body was extruded.

### 2.11. Transcript Quantification by qPCR

A total of 133 oocytes and 71 blastocysts in sheep and 76 oocytes and 28 blastocysts in red deer were processed for RNA extraction and cDNA synthesis and were submitted to qPCR as previously described [9]. Poly(A) RNA was extracted from pools of 10–14 expanded sheep blastocysts and approximately five expanded red deer blastocysts per experimental group (three replicates) using magnetic beads (Dynabeads^®^ mRNA DIRECT™ Micro Kit; Invitrogen^TM^, Carlsbad, California, USA) according to the manufacturer’s guidelines slightly modified by Bermejo-Álvarez et al. [37]. Briefly, each pool of oocytes and blastocysts was resuspended in 50 µL of lysis/binding buffer for 5 min at room temperature. The resulting lysate was then hybridized with 10 µL of prewashed oligo (dT)25 beads. After 5 min of incubation, samples were put into a magnet to remove the lysis buffer while retaining the poly(A) RNA attached to the beads. After hybridization, samples were washed twice in 50 µL of wash buffer A and twice in wash buffer B. Next, the mRNA samples were eluted from the beads with 28 µL of elution buffer (Tris-HCl). Following this, reverse transcription (RT) was performed using the iScript™cDNA Synthesis Kit (Bio-Rad, Madrid, Spain) in a total volume of 40 µL. In the first step, samples were heated to 70 °C for 5 min to denature the secondary RNA structure. First-strand cDNA was generated from the total amount of RNA by adding 4 μL of iScript Reaction Mix (5x), 1 μL of iScript Reverse Transcriptase, and 7 μL of nuclease-free water. The RT reaction was performed at the following conditions: 10 min at 25 °C, followed by 30 min at 42 °C and 5 min at 85 °C.

After RT, the relative abundance of mRNA transcripts was determined by quantitative real-time PCR (qPCR) using a LightCycler 480 II System (Roche Diagnostics, Barcelona, Spain). The qPCR samples consisted of 10 μL of PowerUp™SYBR^®^ Green Master Mix (Thermo Fisher Scientific, Barcelona, Spain), 200 nM each of forward and reverse primers, and 2 µL of cDNA template. Final volume of 20 μL was reached by adding nuclease-free water. The PCR amplification was carried out in the following cycling conditions: 50 °C for 2 min for UDG activation, 95 °C for 2 min, and then 40 cycles of 95 °C for 15 s and 60 °C for 1 min. Finally, a melting curve was produced to confirm the presence of a single gene by heating the samples to 95 °C for 5 s in a ramp rate of 4.4 °C/s, followed by 65 °C for 1 min with a heating rate of 2.2 °C/s, and continuous fluorescence measurement. Two technical replicates per biological replicate and individual gene were evaluated and reactions without any cDNA template (2 μL nuclease-free water) were used as the negative control.

Sixteen separate genes, aldo-keto reductase family 1, member B1 (*AKR1B1*); BCL2-associated X protein (*BAX*); BCL2 apoptosis regulator (*BCL2*); bone morphogenetic protein 15 (*BMP15*); gap junction protein alpha 1 (*GJA1*); glutathione peroxidase 1 (*GPX1*); growth differentiation factor 9 (*GDF9*); fibroblast growth factor 8 (*FGF8*); growth factor 16 (*FGF16*); insulin-like growth factor 2 receptor (*IGF2R*); integral membrane protein 2B (*ITM2B*); nuclear respiratory factor 1 (*NRF1*); DNA polymerase gamma 2, accessory subunit (*POLG2*); SHC adaptor protein (*SHC1*); superoxide dismutase (*SOD2*); and tumor protein p53 (*TP53*), plus an endogenous reference gene (H2A histone family, member Z; *H2AFZ*), were amplified (see Table 1 for all primer information). The comparative cycle threshold (Ct) method [38] was used to calculate the relative transcript abundances with the average of two replicates, and quantification was normalized against that of the endogenous control (*H2AFZ*). Fold changes in the relative abundance of mRNA transcripts were determined for target genes assuming an amplification efficiency of 100% and were calculated using the 2^−ΔΔCT^ method [39].

### 2.12. Statistical Analysis

Melatonin concentration in follicular fluid; oocyte early apoptosis; DNA fragmentation; ROS and GSH content; mitochondrial membrane potential and distribution; meiotic competence; embryo production; and total number of cells in blastocysts were analyzed by factorial ANOVA with the SPSS 24.0 software (IBM, Armonk, NY, USA), after confirming that data were distributed normally and variances were homogeneous. With this analysis, type of ovary storage medium (Melatonin and Control) and the replicate (for embryo production, five replicates; for evaluation of oocyte early apoptosis; DNA fragmentation; ROS and GSH content; mitochondrial membrane potential and distribution; meiotic competence; and total number of cells in blastocysts, three replicates) were considered fixed effects. Additionally, another factorial ANOVA was conducted to examine the relative abundances of mRNA transcripts, with type of ovary storage media and qPCR technical replicate (two replicates) as the fixed effects and the different target genes as the dependent variable. When a significant effect was observed, post hoc comparisons with Bonferroni correction were carried out. Results are presented as mean ± S.E.M.

## 3. Results

### 3.1. Melatonin Addition to the Ovary Storage Medium Increases Melatonin Levels in Follicular Fluid

**Figure 6:**
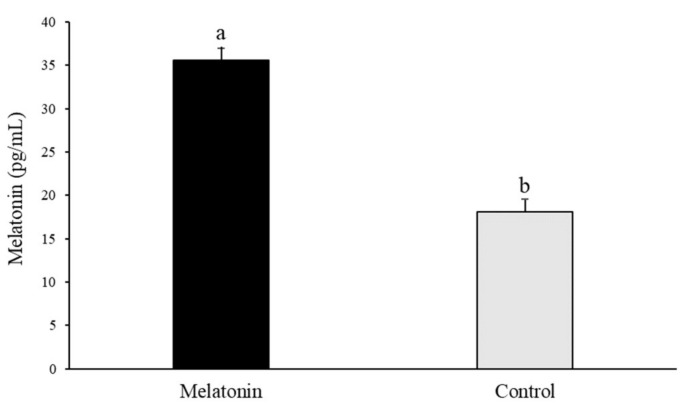
Melatonin concentrations (pg/mL) in samples of follicular fluid collected from sheep ovaries transported in saline solution (Control) and saline solution with 10^−3^ M melatonin (Melatonin); Results are expressed as mean ± SEM; ^a,b^ Different letters indicate differences (*p ≤* 0.05) among treatments.

As shown in Figure 6, melatonin concentration in follicular fluid was increased (*p* < 0.01) when comparing the Melatonin group to the Control group (35.58 ± 1.41 pg/mL and 18.11 ± 1.41 pg/mL, respectively).

### 3.2. Effects of Melatonin on Oocyte Quality

**Figure 7:**
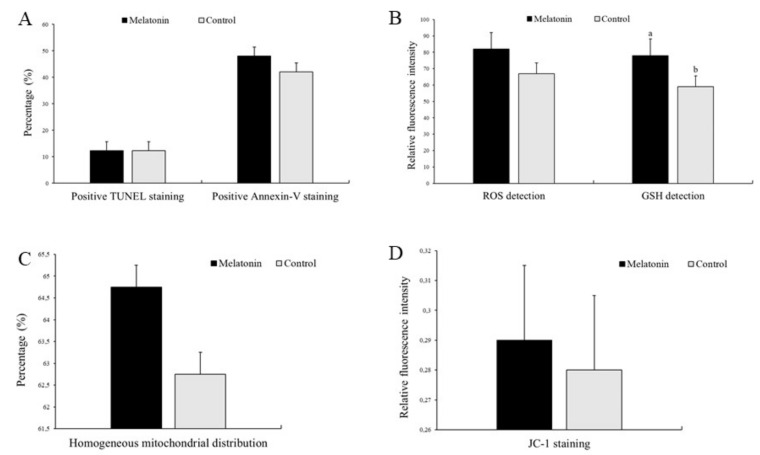
Effect of melatonin supplementation on sheep oocytes’ (**A**) early apoptosis (positive Annexin V staining) and DNA fragmentation (positive TUNEL staining); (**B**) ROS and GSH levels; (**C**) homogeneous mitochondrial distribution; (**D**) mitochondrial membrane potential: red (high membrane potential)/green (low membrane potential) fluorescence ratio (JC-1 staining); Results are expressed as mean ± SEM; ^a,b^ Different letters indicate differences (*p* ≤ 0.05) among treatments.

Early apoptosis and DNA fragmentation assessed by Annexin V and TUNEL staining, respectively, in the Melatonin and Control groups did not show differences (*p* > 0.05; Figure 7A).

Impaired ROS and GSH balance is considered one of the main factors leading to oxidative stress. As shown in Figure 7B, Melatonin and Control-derived oocytes did not exhibit different (*p* > 0.05) levels of ROS. However, melatonin treatment increased (*p* < 0.05) intracellular GSH content as compared to Control (78.01 ± 6.64 and 59.02 ± 6.64, respectively).

Since mitochondria contribute significantly to cellular processes of both ROS production and apoptosis, we examined mitochondrial distribution and membrane potential in oocytes after melatonin supplementation. As shown in Figure 7C, most of the Melatonin and Control-derived oocytes had a normal mitochondrial distribution, with no differences (*p* > 0.05) among groups. Furthermore, the ratio of red to green JC-1 fluorescence did not show differences (*p* > 0.05) between Melatonin and Control oocytes (Figure 7D).

**Figure 8:**
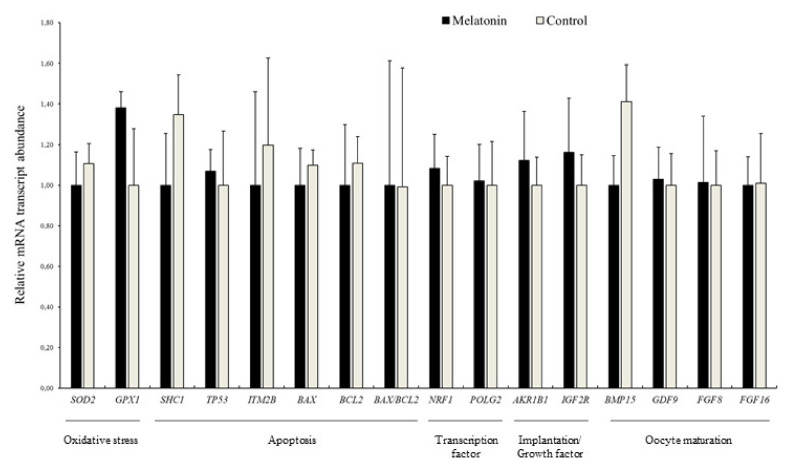
Relative mRNA transcript abundance in Iberian red deer oocytes collected from ovary stored with (Melatonin) and without melatonin (Control); Results are expressed as mean ± SEM.

We also measured the mRNA transcript relative abundance pattern of genes of interest in red deer oocytes. As shown in Figure 8, there were no differences (*p* > 0.05) between treatments in terms of relative mRNA transcript levels. In addition, relative abundance of mRNA transcripts was also analyzed in sheep oocytes, as a complementary assay to deer results, and showed that melatonin supplementation increased the relative abundance of *ITM2B* mRNA transcript compared to Control (*p* < 0.05; Figure S1).

### 3.3. Effects of Melatonin on the Meiotic Competence and Embryo Production

The in vitro meiotic progression on sheep oocytes retrieved from ovaries stored with melatonin and saline solution is summarized in Table S1. The percentage of oocytes that stayed at the GV stage, resumed meiosis (GVBD/MI stage), and reached the MII stage were similar among experimental groups (*p* > 0.05).

The proportion of Iberian red deer oocytes that progressed to the first cleavage stage after in vitro fertilization and the percentage of blastocysts relative to the total number of oocytes was significantly increased (*p* < 0.05) in the Melatonin group compared to Control (Table 2). In sheep, a similar tendency was observed as the percentage of blastocysts relative to the number of cleaved embryos was increased (*p* < 0.05) following melatonin supplementation to the transport medium (Table S2). Moreover, the total cell number in sheep blastocysts was significantly greater (*p* < 0.05) in Melatonin compared with Control sheep blastocysts (108.47 ± 16.83 and 70.84 ± 18.22, respectively).

### 3.4. Effects of Melatonin on the Relative Abundance of mRNA Transcripts in Blastocysts

**Figure 9:**
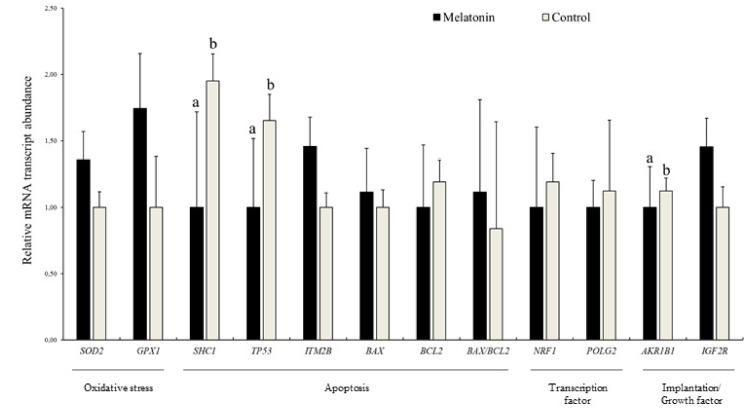
Relative mRNA transcript abundance in Iberia red deer blastocysts produced in vitro from ovary stored with (Melatonin) and without melatonin (Control); Results are expressed as mean ± SEM; ^a,b^ Different letters indicate differences (*p ≤* 0.05) among treatments.

The analyses of two genes involved in ROS detoxification (*SOD2* and *GPX1*), five related to apoptosis (*SHC1*, *TP53*, *ITM2B*, *BAX,* and *BCL2*), two related to mitochondrial function (*NRF1* and *POLG2*), two related to maternal recognition of pregnancy, metabolism, and imprinting (*AKR1B1* and *IGF2R*), one related to GAP junctions (*GJA1*), and a reference gene (*H2AFZ*) were performed in blastocysts derived from both experimental treatments. In Iberian red deer, the relative abundance of *SHC1*, *TP53*, *BCL2,* and *AKR1B1* was less in the Melatonin group compared to Control group (*p* < 0.05; Figure 9). Additionally, mRNA transcript levels in sheep blastocysts showed a lower abundance of *ITM2B* and *BAX/BCL2* ratio, and a greater abundance of *BCL2*, *NRF1,* and *GJA1* in embryos from the Melatonin group (*p* < 0.05; Figure S2).

## 4. Discussion

In vitro-produced Iberian red deer embryos are usually derived from oocytes collected from ovaries obtained at abattoirs or places located far from specialized laboratories. Thus, it usually is a long time between ovary collection and processing in the laboratory. This time interval is a determinant of the quality of oocytes in terms of nuclear maturation and developmental competence after IVM and IVF [10]. During ovary transport to the laboratory, certain factors such as medium composition, storage time, and temperature are crucial to preserve oocyte quality and ensure the success of reproduction technologies such as IVP [35]. Consequently, the present work was conducted to improve the Iberian red deer IVP protocol and its efficiency by modifying the ovary transportation solution with the supplementation of the antioxidant melatonin. To our knowledge, this research is the first to examine the effect of melatonin during ovary preservation on embryo production after IVP, as well as different parameters related to oxidative stress and apoptosis as indicators of the quality of matured oocytes, including the relative mRNA abundance of developmentally important genes in oocytes and blastocysts. When necessary, sheep was used as a research model given the difficulties of working with wild species such as Iberian red deer, since a considerable number of oocytes was required.

After death, the occlusion of blood flow reduces the supply of oxygen and energy to the ovaries and places them under ischemic conditions, resulting in a switch from aerobic to anaerobic cell metabolism [10]. This may induce the accumulation of ROS in the environment surrounding the oocytes and a decrease in their oxidative activity during transportation [41]. In order to complete in vitro development, follicle-enclosed oocytes must remain metabolically active while being transported from the abattoir to the laboratory [41]. Moreover, the female reproductive tissues exhibit higher metabolic rates than other adult organs in mammals [16]. Therefore, it is likely that a rapid increase in ROS production may occur not long after ischemia starts in the ovary. To suppress the detrimental effects of oxygen free radicals in the oocyte, melatonin has been previously used during IVM in several mammalian species [21,22,26,27], however, studies regarding its effect on the preservation of ovaries during shipment are lacking. Despite that, Goodarzi et al. [30] reported a positive effect of melatonin used as a supplement in the preservation solution in sheep. In the present study, greater melatonin concentrations were found in follicular fluid aspirated from sheep ovaries in the Melatonin group compared to the Control group. This finding may be explained by the fact that melatonin is an antioxidant with lipophilic and hydrophilic properties, which allows for a rapid diffusion through cell membranes [42,43]. Besides its physiological role in steroidogenic mechanisms in the follicular fluid, melatonin acts effectively as a free radical scavenger [44], preventing oxidative stress-mediated deterioration of oocyte quality and attenuating the defects of postovulatory aging oocytes [45,46]. Therefore, we hypothesized that the addition of this antioxidant to the transport medium may also have beneficial effects by moderating oocyte oxidative stress during long-term ovary preservation. In fact, our results revealed that the content of GSH of in vitro matured sheep oocytes transported in melatonin was greater than that of the Control oocytes.

Among the main nonenzymatic antioxidants that protect oocytes and embryos against oxidative damage, GSH plays a key role as it appears to reflect the degree of cytoplasmic maturity and quality of oocytes at the end of IVM [34]. Moreover, the addition of melatonin to the IVM medium has been shown to increase the oocyte GSH levels in cattle and pig [47,48], and ultimately promote fertilization and late embryonic development in vitro [49]. However, the pathway through which oocyte intracellular GSH protects from the damage inflicted by long-term ovary preservation remains unknown. Interestingly, our results showed that the supplementation of melatonin resulted in greater rates of embryonic development, indicating that its antioxidant potentiality during ovary storage was sustained throughout embryo cleavage until blastocyst formation. Notably, since the preimplantation embryo does not have the capacity for de novo synthesis of GSH until the blastocyst stage [49,50,51,52], during the early stages of cleavage to morula in vivo, the embryo is protected by the presence of GSH in the maternal reproductive tract [53]. For that reason, early embryos developing in vitro are more sensitive to GSH-depleting agents, even at low levels [52]. Considering this, it can be assumed that the addition of melatonin to the ovary preservation solution may have provided oocytes with large stores of GSH that subsequently stimulated embryo development in vitro.

In this research, the quality of resulting blastocysts in terms of total cell number per blastocyst in sheep was improved by the addition of melatonin to the ovary storage medium. Cell number is a crucial indicator of the development potential of an embryo, as it is directly related to cell cycle progression [54]. The greater numbers of blastomeric cells obtained in Melatonin compared to Control groups may be correlated with the increase in GSH levels observed in matured oocytes, since a positive relationship between oocyte intracytoplasmic GSH content and blastocyst cell number has been previously demonstrated by the addition of several thiol compounds to the IVM medium in goat [55]. Furthermore, our results are also in accordance with the investigation by Goodarzi et al. [30] in which increased blastocyst cell numbers in sheep were observed after melatonin supplementation to the ovary storage medium.

In addition, the use of melatonin in the current study also showed a positive effect on red deer blastocyst quality as presented by the lower abundances of mRNA transcripts for genes involved in oxidative stress and apoptosis. Even though the effect of culture media composition on apoptosis and oxidative stress has been extensively studied in mammalian preimplantation embryos [56,57], information regarding the impact of ovary storage medium on embryo quality is very limited. Notably, SHC adaptor protein (*SHC1*) is linked to increased intracellular ROS generation, cytochrome c release and apoptosis induction, leading to permanent embryo arrest [58,59,60]. Moreover, inhibition of *SHC1* in knockout mice generates a higher ratio of reduced to oxidized glutathione (GSH/GSSG) in embryonic fibroblasts [61]. The encoded protein of the *TP53* gene is a key transcription factor in cell cycle regulation and apoptosis [62]. Oxidative stress activates a *TP53*-transcriptional response, which promotes cell cycle arrest, ROS generation, and apoptosis [63]. Remarkably, the addition of melatonin has been shown to significantly decrease *TP53* transcription levels, promoting subsequent in vitro development by accelerating the G1/S phase transition via the *TP53/TP21* pathway [63]. Aldo-keto reductase family 1, member B1 (*AKR1B1*) is related to implantation failure and/or embryo resorption via PGF2α synthesis, and it may also affect the embryo fate through its involvement in apoptotic pathways [64]. Hence, the greater relative abundance of *SHC1*, *TP53,* and *AKR1B1* mRNA transcripts observed in Iberian red deer blastocysts derived from the Control group in the present study may be reflective of lesser quality embryos. Additionally, the results obtained in sheep point in the same direction since melatonin-derived blastocysts showed patterns of mRNA relative abundance associated with embryos of higher quality.

## 5. Conclusions

In conclusion, the supplementation of melatonin to the ovary storage medium had a positive effect on the developmental competence and blastocyst quality in Iberian red deer. As far as we are concerned, this is the first study to demonstrate a distinctive relationship between ovary preservation media composition and the relative mRNA abundance of several important genes in resulting blastocysts after IVP. Since successful embryo production depends on the maintenance of oocyte viability during transportation of the ovaries over long distances, the use of melatonin for IVP of wild species may successfully preserve the oocyte due to the induction of GSH synthesis. The present findings have important implications for solving the problems associated with the long transport times that usually cannot be avoided when working with wild species such as Iberian red deer. Thus, our research may serve as a basis for future studies on the use of melatonin and other antioxidants during ovary transport to promote the production of greater numbers of red deer embryos of higher quality.

## Figures and Tables

**Table 1:** Details of primers used in this study for qPCR.

Gene	Gene Function	Primer Sequence (5′–3′)	Product Size (bp)	Accession No.
*H2AFZ*	Reference gene	F-ATTGCTGGTGGTGGTGTCAT	147	NM_001009270.1
		R-ACTGGAATCACCAACACTGGA		
*SOD2*	Oxidative stress	F-GCTTACAGATTGCTGCTTGT	101	NM_201527.2
		R-AAGGTAATAAGCATGCTCCC		
*GPX1*		F-GCAACCAGTTTGGGCATCA	116	NM_174076.3
		R-CTCGCACTTTTCGAAGAGCATA		
*SHC1*	Apoptosis	F-GTGAGGTCTGGGCAGAAGC	335	XM_024986737.1
		R-GGTTCGGACAAAAGGATCACC		
*TP53*		F-GACTCTCGTGGTAACCTGCT	91	NM_001009403.1
		R-AATTTTCTTCCTCAGTGCGGC		
*ITM2B* ^a^		F-GTCCCAGAGTTTGCAGATAGTGA	104	NM_001035093.1
		R-GGAATCACATAGCACTTATCCAGGTT		
*BAX* ^b^		F-GTTGTCGCCCTTTTCTACTTTGC	89	NM_173894.1
		R-CAGCCCATGATGGTCCTGATC		
*BCL2*		F-GGAGCTGGTGGTTGACTTTC	518	NM_001077486.2
		R-CTAGGTGGTCATTCAGGTAAG		
*NRF1* ^c^	Transcription factor	F-CTGTCGCCCAAGTGAATTATTCG	67	NM_001098002.2
		R-TGTAACGTGGCCCAGTTTTGT		
*POLG2* ^d^		F-CTTCTGGGAAACTACGGGAGAAC	84	NM_001075191.1
		R-GTAGCCTCTTGTTTACCAGATCCA		
*AKR1B1*	Implantation	F-CGTGATCCCCAAGTCAGTGA	152	NM_001012519.1
		R-AATCCCTGTGGGAGGCACA		
*IGF2R*	Growth factor	F-GCTGCGGTGTGCCAAGTGAAAAAG	201	NM_174352.2
		R-AGCCCCTCTGCCGTTGTTACCT		
GJA1	GAP junctions	F-TGCCTTTCGTTGTAACACTCA	143	NM_174068.2
		R-AGAACACATGAGCCAGGTACA		
*BMP15* ^e^	Oocyte maturation	F-CTACGACTCCGCTTCGTGTGT	69	NM_001031752.1
		R-AGTGCCATGCCACCAGAAC		
*GDF9* ^f^		F-GAAGTGGGACAACTGGATTGTG	71	NM_174681.2
		R-CCCTGGGACAGTCCCCTTTA		
*FGF8* ^g^		F-GGAGATCGTGCTGGAGAACAA	66	NM_001206678.1
		R-GCCATGTACCAGCCCTCGTA		
*FGF16* ^h^		F-CGCTTCGGAATTCTGGAGTT	62	NM_001192777.1
		R-TCCACTCCCCGGATGCT		

F, forward primer; R, reverse primer. ^a–h^ From Ferreira et al. [40].

**Table 2:** Effect of melatonin supplementation during ovary transport on rates of cleavage and blastocyst development in Iberian red deer.

Treatment	*n*	Cleaved Embryo at 48 hpi (%)	Expanded Blastocyst (%)	
			Total	Cleaved
Melatonin	157	40.08 ± 2.51 ^a^	14.06 ± 2.42 ^a^	35.62 ± 9.58
Control	212	31.85 ± 2.45 ^b^	6.63 ± 2.36 ^b^	26.17 ± 9.33

Data expressed as mean ± SEM; Results represent five replicates; ^a,b^ Different letters indicate differences (*p ≤* 0.05) among treatments.

## Supplementary Materials

The following are available online at https://www.mdpi.com/2076-2615/10/5/763/s1. Figure S1: Relative mRNA transcript abundance in sheep oocytes collected from ovary stored with (Melatonin) and without melatonin (Control), Figure S2: Relative mRNA transcript abundance in sheep blastocysts produced in vitro from ovary stored with (Melatonin) and without melatonin (Control), Table S1: In vitro maturation of sheep oocytes retrieved from ovaries stored with and without melatonin, Table S2: Effect of melatonin supplementation during ovary transport on rates of cleavage and blastocyst development in sheep.
