# Supplementary material for: Beneficial Effects of Melatonin in the Ovarian Transport Medium on In Vitro Embryo Production of Iberian Red Deer (Cervus elaphus hispanicus)
Source: Animals (Basel). 2020 Apr 27;10(5):763. doi: 10.3390/ani10050763 (PMC7278470; doi:10.3390/ani10050763)
Supplement: Supplementary file 1 [file animals-10-00763-s001.pdf]

animals MDPI

## Supplementary materials

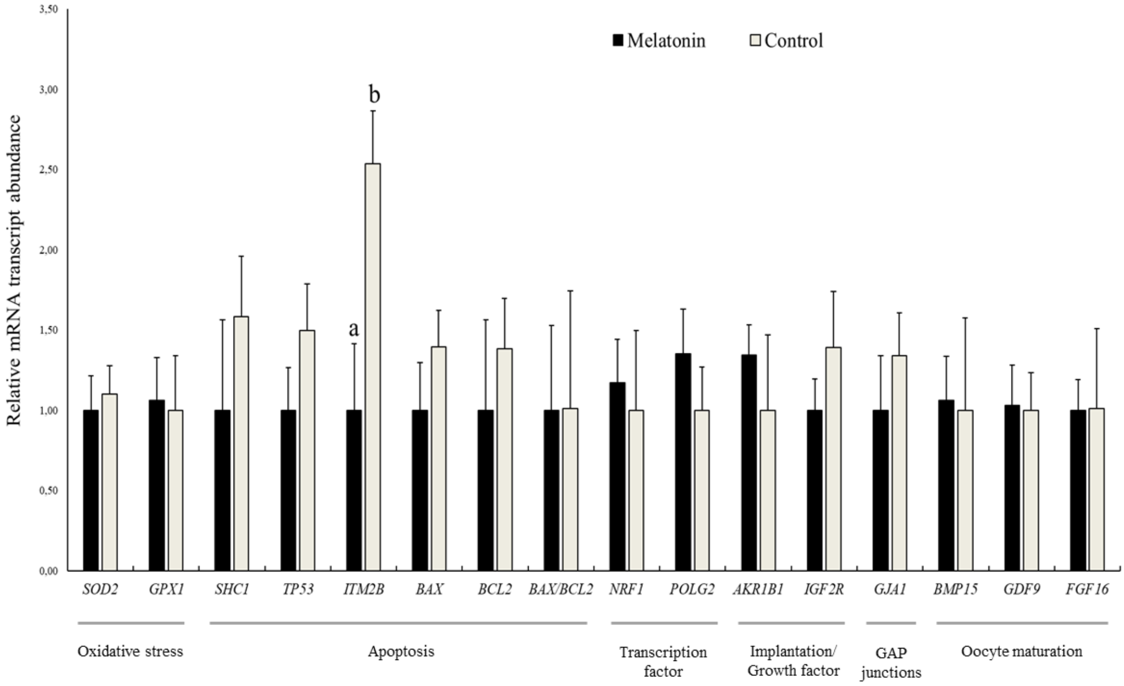


**Figure S1.** Relative mRNA transcript abundance in sheep oocytes collected from ovary stored with (Melatonin) and without melatonin (Control); Results are expressed as mean ± SEM; [a,b] Different letters indicate differences ($p \leq 0.05$) among treatments.

**Table S1.** *In vitro* maturation of sheep oocytes retrieved from ovaries stored with and without melatonin.

| | | Nuclear configuration after IVM | | |
|---|---|---|---|---|
| Treatment | Nº oocytes | GV (%) | GVBD/MI (%) | MII (%) |
| Melatonin | 92 | 11.41 ± 6.39 | 4.19 ± 3.54 | 84.39 ± 6.69 |
| Control | 90 | 8.68 ± 6.39 | 3.12 ± 3.54 | 88.19 ± 6.69 |

Data expressed as mean ± SEM; Results represent three replicates.

**Table S2.** Effect of melatonin supplementation during ovary transport on rates of cleavage and blastocyst development in sheep.

| **Treatment** | ***n*** | **Cleaved embryo at 48 hpi (%)** | **Expanded blastocyst (%)** | |
|---|---|---|---|---|
| | | | **Total** | **Cleaved** |
| Melatonin | 195 | 57.66 ± 4.33 | 26.76 ± 2.35 | 46.86 ± 4.26[a] |
| Control | 195 | 67.97 ± 4.51 | 23.13 ± 2.26 | 30.34 ± 4.10[b] |

Data expressed as mean ± SEM; Results represent five replicates; [a,b]Different letters indicate differences ($P \leq 0.05$) among treatments

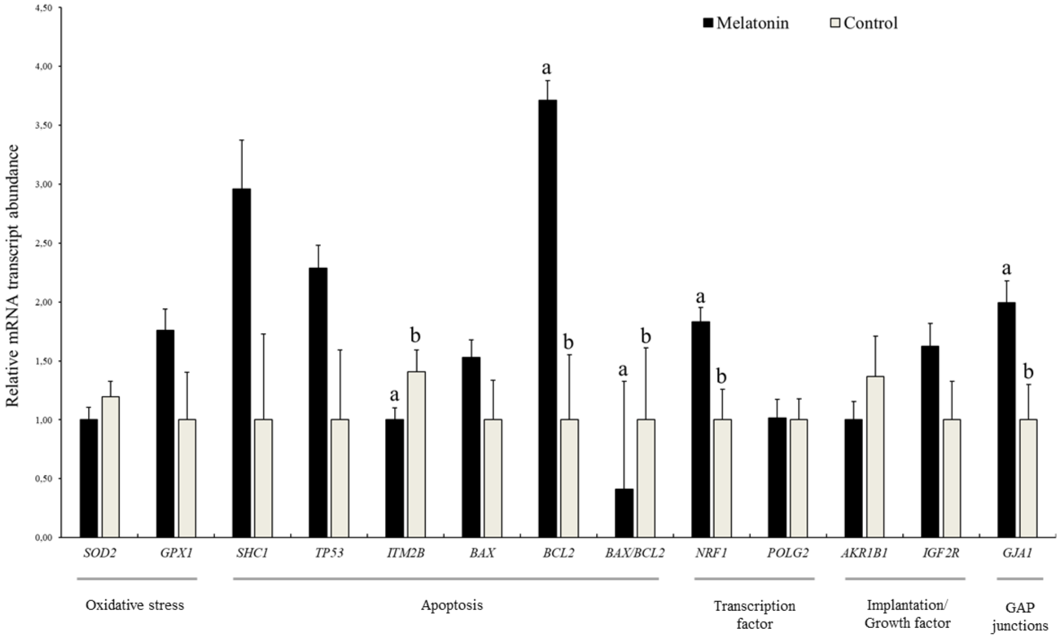


**Figure S2.** Relative mRNA transcript abundance in sheep blastocysts produced *in vitro* from ovary stored with (Melatonin) and without melatonin (Control); Results are expressed as mean ± SEM; [a,b] Different letters indicate differences ($p \leq 0.05$) among treatments
